# Application of Molecular Methods for Carbapenemase Detection

**DOI:** 10.3389/fmicb.2019.01755

**Published:** 2019-08-02

**Authors:** Anastasia Bilozor, Arta Balode, Giorgi Chakhunashvili, Tetyana Chumachenko, Svetlana Egorova, Marina Ivanova, Liidia Kaftyreva, Siiri Kõljalg, Triinu Kõressaar, Olga Lysenko, Jolanta Miciuleviciene, Reet Mändar, Danuta O. Lis, Monika Pomorska Wesolowska, Kaspar Ratnik, Maido Remm, Jelena Rudzko, Tiiu Rööp, Mara Saule, Epp Sepp, Julia Shyshporonok, Leonid Titov, David Tsereteli, Paul Naaber

**Affiliations:** ^1^Department of Microbiology, Central Laboratory, East-Tallinn Central Hospital, Tallinn, Estonia; ^2^Department of Microbiology, Institute of Biomedicine and Translational Medicine, University of Tartu, Tartu, Estonia; ^3^Department of Biology and Microbiology, Riga Stradins University, Riga, Latvia; ^4^Department of Communicable Disease, National Center for Disease Control and Public Health, Tbilisi, Georgia; ^5^Department of Epidemiology, Kharkiv National Medical University, Kharkiv, Ukraine; ^6^Department of Enteric Infections, St-Petersburg Pasteur Institute, Saint Petersburg, Russia; ^7^Department of Bioinformatics, Institute of Molecular and Cell Biology, University of Tartu, Tartu, Estonia; ^8^Department of Bacteriological Laboratory, Kyiv City Clinical Hospital, Kyiv, Ukraine; ^9^Department of Microbiology, Vilnius City Clinical Hospital, Vilnius, Lithuania; ^10^Department of Biohazards and Immunoallergology, Institute of Occupational Medicine and Environmental Health, Sosnowiec, Poland; ^11^Department of Microbiology, KORLAB Medical Laboratory, Ruda Slaska, Poland; ^12^SYNLAB Estonia, Tallinn, Estonia; ^13^Department of Clinical and Experimental Microbiology, Republican Research and Practical Center for Epidemiology and Microbiology, Minsk, Belarus

**Keywords:** carbapenemases, *Klebsiella pneumoniae*, whole genome sequencing, MALDI-TOF carbapenemase assay, luminex carbapenemases assay

## Abstract

This study has evaluated the correlation between different carbapenemases detection methods on carbapenem non-susceptible *Klebsiella pneumoniae* strains from Northern and Eastern Europe; 31 institutions in 9 countries participated in the research project, namely Finland, Estonia, Latvia, Lithuania, Russia, St. Petersburg, Poland, Belarus, Ukraine, and Georgia. During the research program, a total of 5,001 clinical *K. pneumoniae* isolates were screened for any carbapenem non-susceptibility by the disk diffusion method, Vitek 2 or Phoenix system following the EUCAST guideline on detection of resistance mechanisms, version 1.0. Strains isolated from outpatients and hospitalized patients from April 2015 to June 2015 were included. All types of samples (blood, pus, urine, etc.) excluding fecal screening or fecal colonization samples have been represented. In total, 171 carbapenemase screening-positive *K. pneumoniae* isolates (3.42%) were found and characterized. Several methods were used for detection of carbapenemases production, including Luminex assay (PCR and hybridization), whole genome sequencing, MALDI-TOF based Imipenem degradation assay, and immunochromatography testing. Minimal inhibitory concentration determination for Meropenem by agar-based gradient method was also used. Finally, 83 *K. pneumoniae* strains were carbapenemase negative by all confirmation methods (49.4% of all screening-positive ones), 74 – positive by three methods (44.0%), 8 – positive by two methods (4.8%) and 3 – positive by only one method (1.8%). The sensitivity of the tests was 96.3% for Whole genome sequencing and MALDI-TOF assay (both three undetected cases), and 95.1% for Luminex-Carba (4 undetected cases). The most commonly detected carbapenemases were NDM (*n* = 54) and OXA-48 (*n* = 26), followed by KPC-2, VIM-5, and OXA-72 (one case of each). Our results showed that different types of carbapenemases can be detected in the countries involved in the project. The sensitivity of our methods for carbapenemase detection (including screening as a first step and further confirmation tests) was >95%, but we would recommend using different methods to increase the sensitivity of detection and make it more precise.

## Introduction

The rapid worldwide spread of carbapenemase-producing Enterobacterales (CPE) poses a global threat to patient safety and healthcare systems. CPE infections are associated with high mortality, primarily due to delays in the administration of effective treatment and a limited availability of treatment options (European Centre for Disease Prevention, and Control [ECDC], 2018). The increasing percentage of antimicrobial-resistant Enterobacterales is a public health concern of growing importance in Europe and worldwide. Carbapenem-resistant *Klebsiella pneumoniae* is becoming increasingly common in Europe. For *K. pneumoniae*, data from the European Antimicrobial Resistance Surveillance Network (EARS-Net) for 2016 show large differences in the national percentages of carbapenem resistance in invasive (i.e., mostly from bloodstream infections) isolates, ranging from 0 to 66.9% depending on the country (European Centre for Disease Prevention, and Control [ECDC], 2015, 2018). A rapid and accurate method for detecting carbapenemase-producing strains in microbiology lab conditions is one of the measures to prevent the spread of these pathogens.

Nowadays, different methods for detection of carbapenemases are routinely used depending on country policy, financial support, laboratory equipment, workforce, etc. Combination disk testing, biochemical tests, detection of carbapenem hydrolysis with MALDI-TOF, PCR and other Techniques are recommended by guidelines and expert groups (CDC, 2011; EUCAST, 2017). Whole genome sequencing (WGS) is also widely used as confirmation and a reference method (Kaiser et al., 2018).

We describe herein the applicability of different methods to confirm the production of carbapenemase in *K. pneumoniae* strains collected from Northern and Eastern Europe, the aim of the study being to evaluate the correlation between the methods and make some further recommendations.

## Materials and Methods

### Bacterial Strains Collection

Thirty one institutions from nine countries participated in the research project: Finland (1 institution), Estonia (5), Latvia (2), Lithuania (2), Russia, St. Petersburg (9), Poland (2), Belarus (7), Ukraine (2), and Georgia (1).

A total of 5,001 clinical *K. pneumoniae* isolates were screened for any carbapenem non-susceptibility by disk diffusion method, Vitek 2 or Phoenix system following, the EUCAST guideline on detection of resistance mechanisms, version 1.0 (EUCAST, 2013). Strains isolated from outpatients and hospitalized patients between April 2015 and June 2015 were included. All types of samples (blood, pus, urine, etc.) excluding fecal screening or fecal colonization samples were represented.

Different carbapenems were used as a screening option. Carbapenems and their combinations varied from country to country depending on local guidelines and recommendations. Meropenem was used in Belarus, Ukraine and Finland; Meropenem and/or Ertapenem combination in Poland and Lithuania; Meropenem and/or Ertapenem and/or Imipenem combination in Estonia, Georgia, Latvia and St. Petersburg (Russia).

### Identification by MALDI-TOF

Bacterial strains were cultured on Columbia sheep blood-agar plates (Labema, Finland) for 24 h, fresh overnight cultures being used for the tests. Identification was made by MALDI-TOF MS (Bruker Daltonik, Bremen, Germany) using α-cyano-4-hydroxycinnamic acid matrix and standard protocol for identification. Scores of >2 were accepted as final result of species identification.

### MIC Measurement

The minimal inhibitory concentration (MIC) of Meropenem was determined following EUCAST guidelines, which indicates Meropenem as screening carbapenem, offering the best compromise between sensitivity and specificity in terms of detecting carbapenemase-producers (EUCAST, 2017). Fresh overnight cultures were used for 0.5 McFarland preparations, which were further inoculated on Mueller-Hinton agar plates (OXOID, United Kingdom) and MIC test strips (Liofilchem, Italy) were applied. Plates were incubated at 35 ± 1°C for 18 ± 2 h before examination. The interpretation was based on EUCAST Clinical Breakpoint table version 9.0^1^.

### MALDI-TOF Based Carbapenem Degradation Assay

The rapid carbapenem degradation assay was prepared using MBT STAR-BL IMI Kit prototype (Bruker Daltonik, Bremen, Germany). Imipenem is used as a tested carbapenem. Briefly, overnight bacterial cultures including positive and negative controls grown on Columbia blood agar were mixed with reconstituted MBT STAR-BL Antibiotic reagent solution and incubated at 35 ± °C for 30–60 min with agitation (800–900 rpm). The positive control was made with carbapenemase-positive *K. pneumoniae* ATCC BAA 1705 [*Klebsiella pneumoniae* carbapenemase (KPC)-positive] and -negative *K. pneumoniae* ATCC 700603. After incubation, samples were centrifuged for 2 min at 13,000 rpm. One μL of supernatant was applied to a MALDI target plate in 2 MALDI target plate positions each. Air-dried spots were overlaid with 1 μL MBT STAR-BL Matrix solution and thereafter air-dried. Running and interpretation of results were based on the STAR-BL dedicated software module (Bruker Daltonik, Bremen, Germany). Results were presented as graphs showing the carbapenem degradation compared with negative and positive controls. Example of some results visualization can be found in Supplementary Table 1.

### Molecular Carbapenemase Confirmation

Detection of carbapenemase encoding genes used Luminex in-house panel, which included *bla*_IMP_, *bla*_VIM_, *bla*_KPC_, *bla*_GIM_, *bla*_NDM__,_ and *bla*_OXA–48_ genes. DNA was purified from fresh overnight cultures using MagNA Pure 96 DNA and Viral NA Small Volume Kit with MagNA Pure 96 instrument (both Roche, Switzerland), applying the Pathogen Universal 200 protocol (200 μL input; 100 μL DNA elution). Ten μL DNA was used in an in-house PCR protocol at a final volume of 50 μL for direct multiplexed carbapenemase gene amplification.

Luminex^®^ XMAP hybridization technology was applied for detection of the final amplified product and result calling. In short, the in-house selected and validated ssDNA hybridization probes were covalently linked to Luminex^®^ MagPlex carboxylated polystyrene microparticles using the manufacturer’s suggested protocol. One μL PCR antisense product (strand with 5′-biotin) was hybridized to specific pool of MagPlex microparticles and labeled with streptavidin-phycoerythrin (ProZyme, United States). A Luminex^®^ MAGPIX analyzer was used for fluorescence measurement and detection of microparticles. All MFI (median fluorescence intensity) signals above fivefold NTC (no template control) were taken as a positive finding of a specific gene.

The Developed Luminex^®^ in-house method for detection of carbapenemase encoding genes has been validated with samples from Labquality (Finland), HUMB collection (Estonia) and NEQAS EuSCAPE (United Kingdom).

### Whole Genome Sequencing

DNA was extracted from the overnight fresh cultures and sequenced using Illumina HiSeq (Illumina, Inc., United States), with its Nextera XT DNA Library Prep protocol being followed, excluding the library normalization step.

All sequenced genomes were assembled *de novo* using Velvet version 1.2 (Zerbino and Birney, 2008). Before assembly, all reads with low quality were removed after quality control with fastq_quality_trimmer (parameter values –l 40, -t 30) and fastq_quality_filter -(q 25-p 90) from FASTX-Toolkit^2^.

Velvet was run in combination with different parameter values (-cov_cutoff -ins_length -min_pair_count –exp_cov). The most suitable genome assembly was chosen on the basis of the N50 value, the value of the maximum contig length, and the value of the total genome size.

Carbapenemases, extended spectrum beta-lactamases and other beta-lactamase encoding genes were analyzed using ResFinder 2.1 database^3^.

### Immunochromatography Test

In selected cases, i.e., when confirmation tests gave ambiguous result (only one of above described tests was positive), the Coris RESIST-4 O.K.N.V. immunochromatography test (CorisBioconcept SPRL, Belgium) was also used, which detects OXA-48, KPC, NDM, and VIM carbapenemases.

## Results

In total, 171 carbapenemase screening positive out of 5,001 *K. pneumoniae* isolates (3.42%) were found and investigated in detail. The number of screening positive strains by country was: Russia 75, Poland 29, Belarus 23, Estonia and Latvia each 14, Lithuania 9, Georgia 4, Ukraine 2, and Finland 1 (3 isolates that fail sequencing were excluded from further analysis, leaving 168 strains for data analyses).

### MIC Results

**FIGURE 1:**
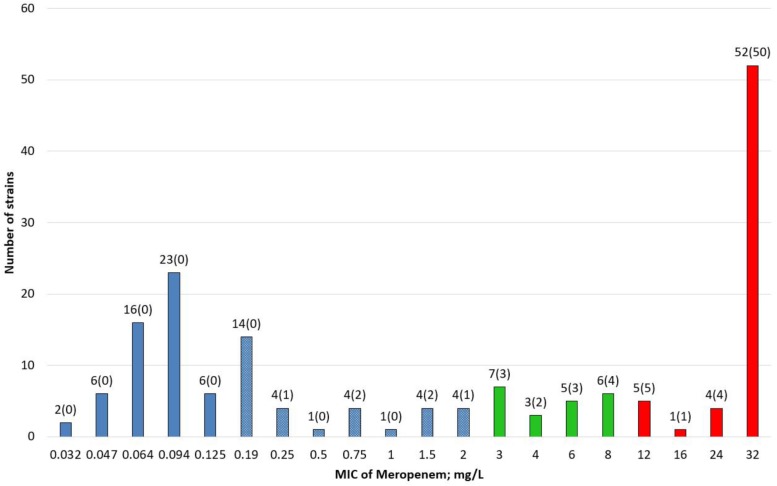
Distribution of Meropenem MIC values in screening-positive *Klebsiella pneumoniae* strains (*n* = 168). Blue boxes: susceptible strains (≤2 mg/L); blue boxes with white dotes: susceptible above screening cut-off (>0.125–≤2 mg/L); green boxes: susceptible, increased exposure (>2–8 mg/L); red boxes: resistant (>8 mg/L). Number of strains (number of strains with carbapenemase gene).

Meropenem MIC results were very diverse, varying from 0.032 to ≥32 mg/L (Figure 1).

According to EUCAST guideline on detectintg resistance mechanisms (EUCAST, 2013), screening cut-off for Meropenem MIC was >0.12 mg/L. MIC values of Meropenem were categorized by EUCAST clinical breakpoints version 9.0, which led to 50.5% susceptible isolates (*n* = 85), susceptible, increased exposure 12.5% (*n* = 21), and resistant 37% (*n* = 62).

### MALDI-TOF Assay

Hydrolysis results corresponding to full enzymatic degradation of Imipenem were detected in 48.2% of isolates (*n* = 81), non-hydrolysed in 50% (*n* = 84), and giving an ambiguous result in 1.8% (*n* = 3). An example of MALDI-TOF assay results can be found in Supplementary Table 1.

Ambiguous results were analyzed as negatives as they could not be confirmed by any other method.

### Detection of Carbapenemase Genes

Carbapenemase encoding genes were detected in 45.6% of the strains (*n* = 78) by Luminex-Carba assay and in 46.2% (*n* = 79) by WGS (Table 1).

**TABLE 1:** Correlation of different methods for detection of CPE and Meropenem MICs in *K. pneumoniae* (*n* = 168).

**Name of the group**	**Number of positive tests**	**Assays positive for carbapenemases**	**Carbapenemase genes detected**	**Number of strains**	**Meropenem MIC range (median), mg/L**
Confirmed CPEs	3	WGS, Luminex-Carba, MALDI-TOF assay	NDM-1	48	1.5–32 (32)
			OXA-48	24	0.75–32 (32)
			KPC-2	1	32
			VIM-5	1	0.75
		Luminex-Carba, MALDI-TOF assay	NDM	2	12; 32
		WGS, MALDI-TOF assay	NDM-1	2	6; 32
	2	WGS, Luminex-Carba	OXA-48	2	0.25; 32
		WGS + Coris	NDM-1	1	0.094
		MALDI-TOF assay + Coris	NDM	1	8
Unclear cases	1	WGS	OXA-72	1	0.047
		MALDI-TOF assay	-	2	0.5; 4
not CPEs	0	All test negative	-	83	0.032–32 (0.094)

### Comparison of Different Methods

MALDI-TOF assay, Luminex assay and WGS were used as methods to confirm carbapenemase production by the 168 *K. pneumoniae* isolates.

Finally 83 *K. pneumoniae* strains were carbapenemase-negative by all three confirmation methods (non-CPEs; 49.4% of all positives), 74 positive by three methods (CPEs; 44.0%), 8 positive by two methods (CPEs; 4.8%) and 3 positive by only one method (unclear cases; 1.8%). The detailed results of different methods, number of strains and Meropenem MICs are presented in Table 1.

As already mentioned, the Coris immunochromatography test was applied to five strains that showed ambiguous result (where only one of above described tests was positive). When unclear, of the strains that were re-tested immunochromatographically, one WGS positive NDM-1 producing strain and one MALDI-TOF assay positive strain were detected as NDM-positive. These two strains were the considered as confirmed CPE.

In the confirmed CPE group, where isolates showed positive results by two methods, two strains were positive by MALDI-TOF assay and Luminex assay, but negative by WGS. There is no satisfactory explanation for the lack of data from WGS since sequence quality had been evaluated. Another two strains showed positive results by MALDI-TOF assay and the NDM gene was found in their genome, but the Luminex assay remained negative. A negative Luminex result might be due to the presence of specific mutations in bacterial genomes, which can lead to absence of matches with the primers being used. In addition, two strains were found to be positive by Luminex, and WGS found the OXA-48 gene, but the MALDI-TOF assay gave a negative. This could be explained by the low activity of OXA enzymes and the need for prolonged incubation time in some cases.

Thus, finally 48.8% of our *K. pneumoniae* strains (*n* = 82; Table 1) were positive by at least two tests and classified as confirmed CPEs. The sensitivity of these tests was 96.3% for WGS and MALDI-TOF assays (both showed three undetected cases) and 95.1% for Luminex-Carba (four undetected cases).

In two strains it remained unclear whether they were carbapenemase producers since they were positive only by MALDI-TOF assay, failing to show any known carbapenemase markers by genotypic or immunochromatography testing. Thus, MALDI-TOF assay gave two possibly false positive cases that were impossible to confirm by other methods. Presumably hydrolysis of Imipenem, which can be detected by MALDI-TOF assay, could be associated with presence of hyperproduction of other enzymes. These strains were positive for the CTX-M-15 gene.

One OXA-72 producing strain was positive only by WGS; since this genotype was not included in the Luminex-Carba and Coris tests, negative results were expected.

The most prevalent detected gene was *bla*_NDM_. NDM-positive isolates (*n* = 54) correspond to 32% (54/168) of all screening positive *K. pneumoniae* strains and 65.9% (54/82) of confirmed carbapenemase producing strains. NDM-producers were found in four participated countries: 1 in Estonia and Lithuania, 4 in Belarus, and 48 in St. Petersburg.

*bla*_OXA–48_ gene was confirmed in 26 isolates, i.e., 15.5% (26/168) of all screening positive *K. pneumoniae* strains, and to 31.7% (26/82) of confirmed carbapenemase producing strains. OXA-48 positive strains were found in Georgia (*n* = 4), Belarus (*n* = 7), and St. Petersburg (*n* = 15).

Also one KPC-producing and one VIM-producing *K. pneumoniae* strains were found in St. Petersburg and Latvia, respectively. One OXA-72 producing strain was found in Belarus.

More than half of isolates screening positively (53.6%; 90/168) determined by the countries themselves gave negative results by the Luminex-Carba assay. No carbapenemase producers were found in Poland, Ukraine, and Finland by all the methods.

Among the non-CPE strains, Meropenem MIC varied from 0.032 to 32 mg/L, with a median of 0.094 mg/L. High MIC values could be associated with another resistance mechanism than carbapenemase production. Confirmed CPEs showed MIC values of Meropenem from 0.75 to 32 mg/L, with a median of 32 mg/L, which corresponds to a resistant result by EUCAST clinical breakpoints (version 9.0). The median of Meropenem MIC for the group of OXA-48 and NDM confirmed strains was 32 mg/L. A significant difference (*p* ≤ 0.0001) in Meropenem MIC values was seen between carbapenemase producing (OXA-48 positive or NDM positive strains) and non-producing strains. There was no significant difference between OXA-48 positive and NDM positive isolates (*p* = 0.4605).

Among isolates with reduced susceptibility to Meropenem 90% (*n* = 75) hydrolyzed Imipenem in MALDI-TOF assay; 8.4% (*n* = 7) showed no hydrolysis and 1 ambiguous result was observed. Meropenem-sensitive strains (*n* = 85) showed no hydrolysis in 90.6% (*n* = 77), complete hydrolysis in 7.1% (*n* = 6) and 2.3% – ambiguous result (*n* = 2). For detailed results see Table 2.

**TABLE 2:** Correlation between MALDI-TOF assay and Luminex assay results.

	**Negative by Luminex-Carba**	***bla*_KPC_ gene**	***bla*_VIM_ gene**	***bla*_OXA–48_ gene**	***bla*_NDM_ gene**
Hydrolyzed result (*n* = 81)	5	1	1	24	50
Non-hydrolyzed result (*n* = 84)	82	-	-	2	-
Ambiguous result (*n* = 3)	3	-	-	-	-

Among the hydrolyzed isolates in the MALDI-TOF assay (*n* = 81), different carbapenemase encoding genes were detected in 94% (*n* = 76), and 6% (*n* = 5) have negative Luminex-Carba results.

Ambiguous results by the MALDI-TOF assay – impossible to interpret as positive or negative – occurred in three cases. These strains were negative regarding any carbapenemase gene by PCR, and their Meropenem MIC varied from 0.094 to 3 mg/L.

Comparing the prevalence of other *bla*-genes in different groups (carbapenemase positive and negative), no significant differences were found for most of the genes, except *bla*_DHA–1_ (AmpC type beta-lactamase) present only in carbapenemase negative strains (*p* < 0.0001). Most of positive strains had CTX-M ESBL genes, and CTX-M-15 dominated in this group. Another common *bla*-gene was SHV-188. The presence of different *bla* genes is shown in Table 3.

**TABLE 3:** Presence of other beta-lactamase genes in screening positive strains arranged by carbapenemase groups and countries.

**Carbapenemase genes detected**	**Number of strains**	**Country of origin (number of strains)**					
			**Other beta-lactamase genes by Bush -Jacoby groups (number of strains)**				
			
			**1 (AmpC type)**	**2be (ESBL)**	**2b**	**2d**	**unknown**
				CTX-M-15 (45);			
NDM/NDM-1	54	RU (48); BY (4);		CTX-M-3 (3);	TEM-1A (3);	OXA-1 (51);	SHV-188 (54); TEM-
		LT (1); EE (1)		CTX-M-124 (1)	TEM-98 (2)	OXA-9 (15)	150 (1)
OXA-48	26	RU (15); BY (7);				OXA-1 (20);	SHV-188 (25); SHV-53
		GE (4)		CTX-M-15 (24)	TEM-1A (11)	OXA-9 (3)	(1)
KPC-2	1	RU				OXA-9	SHV-123; TEM-198
VIM-5	1	LV		CTX-M-15			SHV-188
OXA-72	1	BY					SHV-188
Unclear^*^	2	RU		CTX-M-15 (2)		OXA-1 (1)	SHV-188 (2);
		PL (29); EE (13);		CTX-M-15 (63);			SHV-188 (77);SHV-
		LV (13); BY (9);		CTX-M-5 (2);		OXA-1 (53);	112 (3); TEM-150 (2);
Negative	83	RU (9); LT (8);	DHA-1 (18)	CTX-M-3 (1);	TEM-1 (53)	OXA-9 (27)	SHV-122 (1); SHV-187
		UA (1); FI (1);		CTX-M-14 (1)			(1)

^*^Positive only by MALDI-TOF assay; no carbapenemase detected by other methods.

## Discussion

We have compared different confirmation methods of detecting carbapenemase production by *K. pneumoniae* strains from nine European countries. Overall, ∼1% of all clinical Enterobacterales strains had decreased susceptibility to carbapenems, among them *K. pneumoniae* predominated. Only in half the cases of reduced carbapenem susceptibility, carbapenemase gene/carbapenemase production was confirmed. The low prevalence of carbapenemases can be explained by using screening methods with different specificities on the one hand and high prevalence of other resistance mechanisms on the other. Some labs used reduced susceptibility to Ertapenem in their screening, which is the most sensitive carbapenem for detecting hydrolysis and other resistance mechanisms, but at the same time it is the least specific marker for carbapenemases detection (EUCAST, 2017).

Other mechanisms, such as hyperproduction of AmpC or other ESBL enzymes in combination with porin loss or efflux, can reduce susceptibility to carbapenems (EUCAST, 2017). In 22% of carbapenemase negative strains, AmpC type beta-lactamase gene was found (not found in any carbapenemase positive strain). Others had different ESBL or different *bla*-genes that can be responsible for reduced susceptibility to different beta-lactam antibiotics, and to carbapenems in combination with other mechanism also.

Whole genome sequencing can be used in the routine laboratory workflow for typing pathogens during hospital outbreaks (Rossen et al., 2018). Different resistance genes to antibiotics, disinfectants and other chemicals used in hospitals could be investigated. However, WGS implementation to clinical workflow is a big challenge for different levels of diagnostic facilities. Long turnaround time, high costs and the need for highly qualified bioinformaticians make this process very complicated. In addition, quality assurance procedures and validation procedures are not sufficiently standardized and are far from being straightforward (Ellington et al., 2017; Rossen et al., 2018). Under the conditions of our investigations, sensitivity of WGS was very high (96.3%). Using this method as a reference to others, we could confirm one OXA-72 positive strain, which was out of scope of the Luminex-Carba and Coris tests.

MALDI-TOF assay was a quick and reliable method (sensitivity 96.3%) of detecting carbapenemases, but with limited discriminatory power in specifying the enzyme type. The carbapenem used in a kit by the manufacturer was Imipenem, whereas Meropenem is described as a most sensitive for screening carbapenemases. The use of MALDI-TOF assay also requires specific software.

The Luminex-Carba assay had high sensitivity (95.1%), but the main drawback of the method is the possibility to detect only genes specified by the primers being used, which was the reason of missing one OXA-72 positive strain. PCR-based techniques are more widely spread in clinical lab conditions, and frequently used as a reference confirmation method to phenotypical tests in terms of resistance mechanisms detection.

The Coris immunochromatography test can detect carbapenemases where there is a lack of other expensive methods and time, i.e., it is quick and affordable. The sensitivity of the method (94.9%) has been described in our previous study (Naaber et al., 2018). The limitation is the same as in many commercial PCR kit, i.e., it can test for only a limited number of enzymes.

In summary, our results show that different types of carbapenemases can be detected in the countries involved in this project. The sensitivity of the different methods used for carbapenemase detection, including screening as a first step and confirmation tests, was > 95%, and we would therefore recommend using different methods to increase the sensitivity of detection and make it more precise.

## Data Availability

The datasets generated for this study can be found in the NCBI GenBank (LINK- will be available prior to publication).

## Ethics Statement

Approval was not required as per the local legislation. Institutions used only samples sent for routine diagnostics, no additional sampling was performed. No patient data was used, all strains were coded and processed anonymously (it is impossible to identify any patient by strain number).

## Author Contributions

ABi is a principal investigator and contributed to the preparation of manuscript. ABa and MS are enilabAMR and BARN county coordinator in Latvia, contributed to the data and strain collection, and critical reading of the article. GC and DT are enilabAMR and BARN county coordinator in Georgia, contributed data and strain collection, and critical reading of the article. TC and OL are enilabAMR and BARN county coordinator in Ukraine, contributed to the data and strain collection, and critical reading of the article. SE and LK are enilabAMR and BARN county coordinator in Russia, contributed to the data and strain collection, and critical reading of the article. MI is an enilabAMR and BARN technical coordinator and contributed to the data preparation of the article. SK is an ARMMD coordinator and has contributed to the preparation of manuscript. TK WGS contributed to the data analyses. JM is a enilabAMR and BARN county coordinator in Lithuania, contributed to the data and strain collection, and critical reading of the article. RM contributed to the critical reading of the manuscript. DL and MW are enilabAMR and BARN county coordinator in Poland, contributed to the data and strain collection, and critical reading of the article. KR contributed to the development of Luminex assay. MR contributed to WGS data analyses and preparation of the manuscript. JR contributed to the in-house tests development and performance. TR contributed to the characterization of the strains and was responsible for culture collection. ES was responsible for sequencing and preparation of manuscript. JS and LT are enilabAMR and BARN county coordinator in Belarus, contributed to the data and strain collection, and critical reading of the article. PN is a project coordinator and supervisor, and contributed to the preparation of the article.

## Conflict of Interest Statement

The authors declare that the research was conducted in the absence of any commercial or financial relationships that could be construed as a potential conflict of interest.

## Footnotes

1 http://www.eucast.org/clinical_breakpoints/

2 http://hannonlab.cshl.edu/fastx_toolkit/index.html

3 https://cge.cbs.dtu.dk/services/ResFinder/
