# Supplementary material for: Application of Molecular Methods for Carbapenemase Detection
Source: Front Microbiol. 2019 Aug 2;10:1755. doi: 10.3389/fmicb.2019.01755 (PMC6687770; doi:10.3389/fmicb.2019.01755)
Supplement: Supplementary file 1 [file Table_1.DOCX]

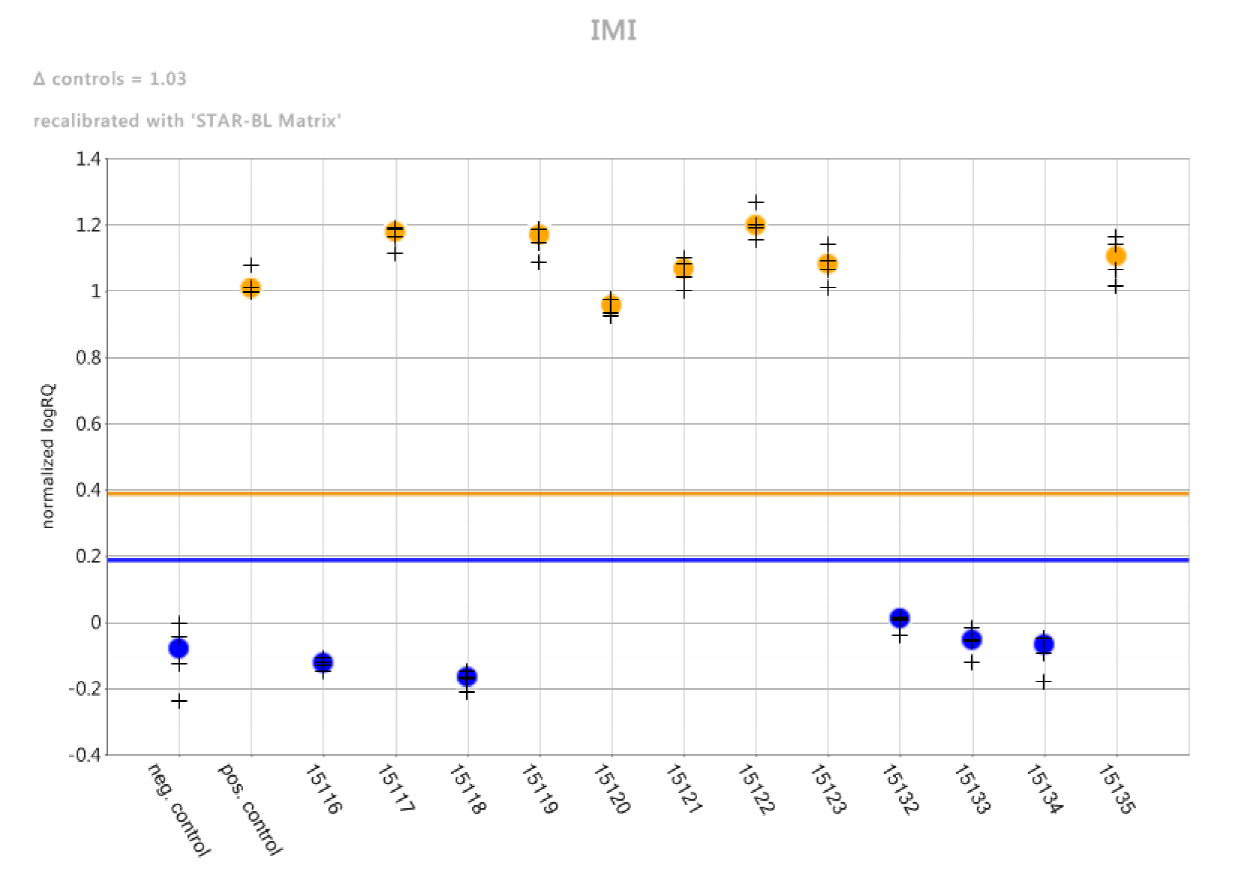


Supplement 1. Example of MALDI-TOF assay results to some strains including positive and negative controls: blue marks below the blue line correspond to non-hydrolysed results, yellow marks above yellow line – hydrolysed results.
